# Microbiome applications for pathology: challenges of low microbial biomass samples during diagnostic testing

**DOI:** 10.1002/cjp2.151

**Published:** 2020-01-15

**Authors:** Caitlin A Selway, Raphael Eisenhofer, Laura S Weyrich

**Affiliations:** ^1^ Australian Centre for Ancient DNA, Department of Molecular and Biomedical Science, University of Adelaide Adelaide SA Australia; ^2^ Department of Anthropology Pennsylvania State University University Park PA USA; ^3^ Huck Institutes of the Life Sciences Pennsylvania State University University Park PA USA

**Keywords:** pathology, microbiome, diagnostic testing, microbiota, personalised medicine, low biomass, contamination, clinical microbiology

## Abstract

The human microbiome can play key roles in disease, and diagnostic testing will soon have the ability to examine these roles in the context of clinical applications. Currently, most diagnostic testing in pathology applications focuses on a small number of disease‐causing microbes and dismisses the whole microbial community that causes or is modulated by disease. Microbiome modifications have already provided clinically relevant insights in gut and oral diseases, such as irritable bowel disease, but there are currently limitations when clinically examining microbiomes outside of these body sites. This is critical, as the majority of microbial samples used in pathology originate from body sites that contain low concentrations of microbial DNA, including skin, tissue, blood, and urine. These samples, also known as low microbial biomass samples, are difficult to examine without careful consideration and precautions to mitigate contamination and biases. Here, we present the limitations when analysing low microbial biomass samples using current protocols and techniques and highlight the advantages that microbiome testing can offer diagnostics in the future, if the proper precautions are implemented. Specifically, we discuss the sources of contamination and biases that may result in false assessments for these sample types. Finally, we provide recommendations to mitigate contamination and biases from low microbial biomass samples during diagnostic testing, which will be especially important to effectively diagnose and treat patients using microbiome analyses.

## Introduction

Existing pathology techniques currently survey small numbers of disease‐causing microbes by applying Koch's postulates. Koch's postulates explain the relationship between a single culturable microbial isolate and a disease, but these postulates only explain a small number of microbial related diseases 1. The significance of more microbially complex diseases is now well appreciated. In most cases, human genetics and single microbes do not explain the full disease pathology, such as urinary tract infections 2 or periodontitis 3. Such diseases result from polymicrobial infections or complex interspecies interactions that can only be understood by looking at the microbiome – an ecosystem of complex microbial communities – in conjunction with human genetics and transcriptomics 4, 5. The human microbiome consists of diverse microbial communities (microbiota) that live on external and internal surfaces of the human body 6, as well as the genetic content and environment of these microbes. Disruptions to the microbiota, through factors such as diet, environment, and medical treatment (i.e. antibiotics), can alter the microbiota structure and contribute to disease 7. It is now known that numerous non‐infectious diseases, including inflammatory bowel disease 7, asthma 8, and neurological disorders 9, are associated with alterations in the microbiome. However, current techniques used in pathology cannot readily detect and characterise ecosystem shifts within these communities, as well as unknown or unculturable pathogens, which can impact the ability to accurately diagnose some diseases.

Most ongoing microbiome research is focused on areas of the body that have high concentrations of microbes (i.e. gut or mouth). However, most samples used for pathological screening have fewer microbes and are known as low microbial biomass samples. Low microbial biomass samples can be easily overwhelmed by contamination from background DNA and are more prone to technical biases, such as over‐amplification during PCR 10, 11, 12, 13. As investigations into low microbial biomass body sites increase, it is vital that new protocols and techniques are applied to minimise the effect that contamination and biases have on these samples and that the limitations are fully understood when developing diagnostic tools based on the results. Here, we review the current techniques used in diagnostic testing and discuss how microbiome assessments can be incorporated into pathology in the future, as many of these techniques have not yet been successfully developed in a diagnostic setting. Lastly, we discuss issues during analysis of low microbial biomass samples in past studies, while highlighting the sources of contamination and biases, and review techniques that can be applied to minimise these confounding factors as microbiome tools are developed moving forward.

## Assessing and treating diseases in pathology: The present and the future

### Limitations of current diagnostic testing for single pathogens

Current diagnostic testing is continuously improving with technological advancements, allowing for more accurate detection of diseases and providing information for precise treatment options. Most current practices used to identify a microorganism during an infection involve collecting a sample (e.g. swab, bodily fluid, or tissue) of the infected area. The sample is then prepared for microscopic analysis, culture, or a PCR‐based method to identify a single or small range of pathogenic species that were previously characterised to cause those disease symptoms 14. However, identifying specific disease‐causing microbes can be a time‐consuming process 15, during a period when patients are potentially left untreated or are administered treatments that are not targeted for a specific condition, such as broad‐spectrum antibiotics 16. In addition, this technology cannot identify unknown pathogens or diverse mixtures of microbes, resulting in delays when investigating rapidly emerging, novel, or unculturable pathogens 17, 18. Lastly, contamination from sample collection and laboratory technicians is not adequately addressed at present. There seems to be no national or international standard that specifically standardises sample collection, with the exception of gloves to be worn to protect the technician. This dismissal of protecting the integrity of the sample could lead to the inadvertent introduction of additional microbes into the sample, which can modify or bias results, and could ultimately result in incorrect diagnoses and treatment of patients 18. New approaches based on sensitive, high‐throughput techniques that investigate unknown microbes, or the microbial community as a whole, are required to mitigate some of these issues and better identify disease sources and complications.

### Benefits of microbiome analysis for pathology

Current research efforts assess changes to the microbiome to understand disease pathologies on a case‐by‐case basis. Approaches used for microbiome analysis can assist in characterising both communicable (infectious) and non‐communicable (non‐infectious) diseases, with non‐communicable conditions being more prevalent in high‐income countries 19. Microbiome analysis provides clinicians with the ability to practice precision medicine, especially in unique or unsolved cases, as it will enable the identification of specific unknown pathogen(s) and the ability to monitor the microbiota and microbiome through time, in relationship to disease status and treatment 4, 20, 21. A current set of procedures to explore microbiomes in diagnostic testing could be: sample collection from the affected area; nucleic acid extraction; sequencing library preparation; sequencing using high‐throughput sequencing (HTS) approaches; and finally, reconstruction of the microbiota and/or their functions using high‐throughput computing resources 22. Several types of HTS can be employed 23, including amplicon based sequencing (targeting one ‘fingerprint’ or ‘barcode’ gene to identify the microbiota present, such as the gene encoding 16S ribosomal RNA) 22; shotgun metagenomic sequencing (assessing a random sampling of DNA from the biological sample to reconstruct microbial genomes and functions of known, new, or under characterised species/strains in the microbiome) 24; and metatranscriptomics (examining the actively transcribed genes using RNA based sequencing approaches) 25. These approaches can aid in the identification, function, and activity levels of known and novel species/strains that contribute to infectious diseases 4, 26, 27, which is important for diagnosis and treatment, but also critical for the downstream development of new rapid and cost effective techniques to readily detect these pathogens. Lastly, understanding the functions of the microbiota and how these functions are utilised is also essential to understand the underpinning mechanisms of disease, within both infectious and non‐infectious conditions. The human microbiome typically contributes over 3 million genes in every single human, which is approximately 150 times more genes than the human genome 28. This volume of information is not routinely assessed in current diagnostic testing and could inform more effective treatment strategies or identify unknown infection dynamics. Below, we discuss in detail how microbiome testing can provide additional information when diagnosing communicable and non‐communicable diseases in the future.

1. *Identifying infectious disease from new or unknown pathogens*

While the most common approach to diagnose an infectious disease is to test for a single pathogen, this approach does not explore unknown or unidentifiable pathogens. Metagenomics analysis can be used to reconstruct the genomes of novel or unknown pathogens by comparison to a distantly related species or strain, to assemble genomes of unknown species from a sample using *de novo* approaches, to reconstruct genomic information, or to quantify levels of a taxon that is present in one location but absent or lower in another, perhaps providing information about an opportunistic pathogen. For example, in one report, a patient suffered with chronic meningoencephalitis for 3 years with no known disease aetiology using standard pathology tests 4. An assessment of the microbiome (metagenomics) revealed that the Cache Valley virus (not known to cause meningoencephalitis) was responsible for the disease 4. In concert with metagenomics analysis, host transcriptomics can also be used to identify if a disease is caused by an infection 4, 29 by examining the active transcription of immune genes activated during infection. Certain host genes are transcribed when fighting an infectious disease, and these transcripts, or their absence, can be detected using current transcriptomic approaches, providing additional clues to the type of infection 29. Quantifying the level of these particular transcripts can also distinguish between an infectious and non‐infectious disease 30, 31.

2. *Co‐infections or diverse poly‐microbial infections*

Diagnostic testing is typically limited to one potential pathogen per test; however, several diseases can manifest as co‐infections or poly‐microbial infections, where multiple microorganisms contribute to the disease. Poly‐microbial infections, such as those observed in the urinary and respiratory tracts, are often difficult to treat due to the interactions between different microbes, so understanding the mechanisms that underpin these infections could improve treatment strategies 32, 33. Amplicon or metagenomic analysis can be applied to identify numerous pathogens simultaneously or, potentially, assess levels of opportunistic pathogens, if a healthy sample has been taken previously.

3. *Function(s) of disease‐causing pathogen(s)*

Assessing specific pathogen functions is essential to identify the correct treatment option(s), especially in cases where the first line of treatment is ineffective. This is typically done by screening a cultured pathogen against different types of antimicrobials. However, the resistance for these antimicrobials is encoded in the genome of each microbe, which contains specific genes for individualised functions. For example, some pathogens carry antimicrobial resistance genes that can provide broad spectrum or very specific antibiotic resistance 34. Metagenomic sequencing could identify which (if any) antimicrobial resistance genes a microbe has and provide information to advise which antibiotic would be the most specific and effective 35. In addition, metatranscriptomics – examination of the RNA in the microbiome – could provide key information on the microbes that are actively playing a role in drug metabolism 36 or antibiotic resistance 37. This information could then be used for better and more targeted treatment, which is especially critical in the light of rising antibiotic resistance.

4. *Microbiome functions for non‐infectious diseases*

Critically, microbiome assessment may prove to be most useful during the diagnosis and treatment of non‐infectious diseases. Autoimmune, allergic, and inflammatory disease are on the rise in industrialised countries 38 and, in many cases, their primary causes remain unclear 39, 40, 41. For example, inflammatory bowel disease has recently been linked to disruptions in the gut microbiota, and there is research underway to determine whether faecal microbiota transplants can be an effective treatment 41. Other microbiome transplants have also been recently suggested, opening the door for different types of microbiome transplantation. An amplicon or metagenomics‐based approach could be utilised to assess the donor's or patient's microbiota or microbiome and identify which specific transplant donor might be best, which microbial functions are missing from the patient's gut, or which probiotic strategies may be the most useful 41. Overall, characterising and assessing the microbiome can be another tool to help identify non‐infectious disease causes or complications and inform more specific and effective treatments.

### Implementing microbiome analysis in pathology: Challenges of working with low‐biomass samples

While there are many advantages to investigating the microbiome using HTS, the widespread implementation of such technologies in a medical context is still limited by certain factors. Clinicians, pathologists, and bioinformaticians require training to properly collect, process, analyse, and interpret microbiome samples, and minimum standards for laboratory analysis and reporting are needed to ensure robust diagnosis and treatment. In some cases (e.g. non‐infectious diseases), the microbial communities and functions linked to disease are still being described; thus, only hypotheses related to causality and function of non‐infectious diseases are described 42, 43. The bioinformatics and sequencing technologies needed to completely describe non‐infectious diseases are not adequate at present; therefore, more research still needs to be undertaken before the microbiome can be analysed routinely in diagnostics 42, 43. Several issues for implementing reliable microbiome analysis in diagnostic testing are already known and need to be addressed in the diagnostics field as microbiome testing is developed, implemented, and employed. This is largely due to the fact that many of the samples commonly screened in diagnostic testing contain a low microbial biomass.

There are numerous areas of the body that are now considered to be of low microbial biomass but were originally thought to be sterile. For example, research has shown that microbes colonise and perform critical functions within the lungs, albeit at low concentrations 44. Other examples of low microbial biomass body sites include skin 45, blood 46, urine 47 and tissue 48 – all of which are typical samples collected for diagnostic tests. These samples typically have small numbers of microbial cells (100–10000 cells/mL) 49, 50 and are more difficult to examine than those of high microbial biomass. Even in widespread microbiome research today, common microbiome protocols 51, 52, 53 are not optimised for low microbial biomass samples. Recent research has provided improvements in low biomass laboratory and analysis protocols 54, 55, but there is still room for further improvement.

The most significant issue in examining low biomass samples is contaminating, or exogenous, DNA (i.e. DNA from sources other than the sample of interest), which is unintentionally introduced during collection and processing of biological samples 10, 13, 48, 56. Contaminating DNA originates from numerous sources, including cells or small fragments of DNA from the same environment, sampling equipment, laboratory reagents and equipment, technicians, and so on 10, 12, 13. Although sterilisation lyses and kills microbial cells, their DNA can be broken up into smaller fragments, which can still be extracted and amplified by PCR. Unsurprisingly, HTS is more sensitive than traditional culture methods in detecting contamination, and if no controls are used to identify contamination, the results may be confounded 57. Using these HTS approaches, contamination has driven spurious conclusions and drastically altered several reported biological discoveries in the literature 56, 58, 59. Especially in low microbial biomass samples, contamination and bias from contaminants has already had severe impacts on the fidelity of reported results 12. As the application of microbiome research shifts towards clinical use, contamination needs to be monitored and accounted for to prevent patient misdiagnoses.

### Lessons from past low biomass microbiome studies

Despite the potential benefits, some researchers working with low microbial biomass samples do not use the necessary precautions to control or limit contamination. Initial studies of the gut microbiome (a source of high microbial biomass) were less prone to contamination, as contaminating DNA was negligible compared to the endogenous microbial DNA (DNA belonging to the sample of interest) present in the sample, and thus downstream analyses were not severely impacted 60. Researchers then followed similar protocols to examine low microbial biomass samples, including placental tissue 58, nipple aspirate 61, and tumours 62, 63, overlooking the possible negative impacts of contamination. This resulted in generating data from low microbial biomass samples that were overwhelmed by the amount of exogenous DNA relative to the endogenous DNA and consequently, contamination was falsely reported as a true result in some studies 56, 58, 59. These past errors are lessons for the future, but we need to ensure they are not repeated, especially when applying microbiome techniques in diagnostic testing.

Lack of controls in microbiome analysis of medical samples has already caused issues within the field. For example, initial investigations into the placenta microbiome sought to answer many questions about infant development and preterm birth during pregnancy 58. However, an initial study failed to use the necessary precautions to monitor and minimise contamination. Non‐template controls were introduced during the extractions, but only a subset were sequenced. Sequences found in the negative controls were not critically compared to those from the biological samples. Additionally, no environmental controls were collected or analysed, preventing the detection of environmental contaminants (e.g. microbes in the air, from the technician, or on sterile dissection equipment). Lastly, the limit of detection was not established, preventing the researchers' ability to determine if a reliable signal could be detected. Contaminant microbial species could therefore not be correctly identified or assessed within the placenta samples to determine if the microbial signature from the placenta was truly endogenous. In 2016, Lauder *et al* 56 replicated the study of the placenta microbiome and found that the microbial placenta profiles resembled those of extraction blank control (EBC) samples and air samples, suggesting that the placenta was likely sterile and did not contain a diverse microbial signal. Since then, many other well‐controlled studies have further supported the lack of a placenta microbiome 59, 64, 65, 66, 67. These studies highlight the importance of having robust protocols and controls to avoid spurious conclusions.

### Sources of contamination and biases in low microbial biomass samples

**Figure 1:**
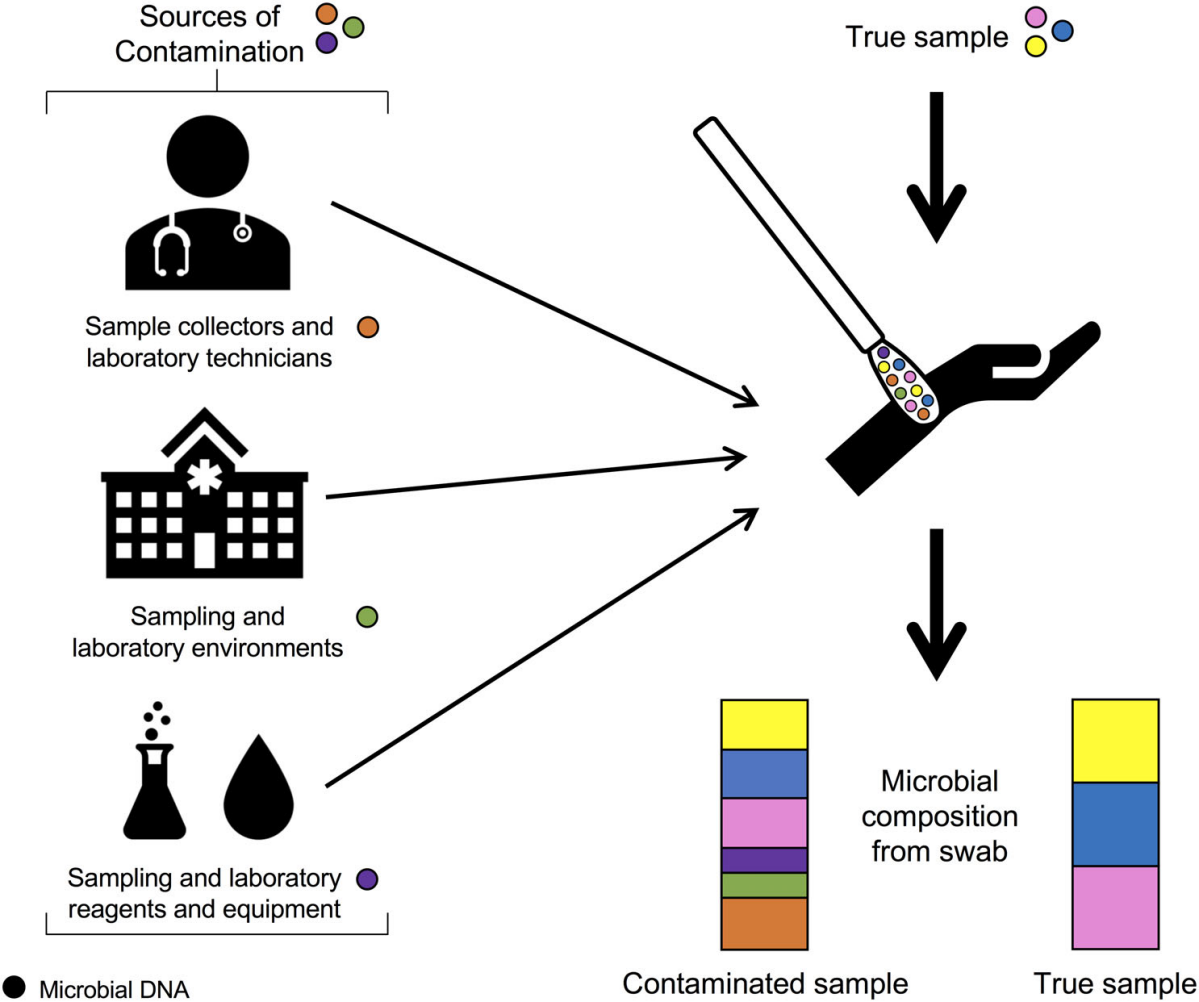
Microbial DNA from sample collectors and laboratory technicians, the environment, and reagents and equipment can contaminate pathological samples, which can distort the microbial profile of the sample.

To better control for contamination and biases, a solid understanding of when and how these factors arise is needed. While contaminating DNA and biases can be introduced at any stage in the sample preparation and analysis process, three predominant sources originate from sampling procedures and the laboratory: (1) doctors, nurses, technicians, and so on; (2) environments; and (3) reagents and equipment (Figure 1). More recently, there has been an increased awareness of contamination and biases introduced into low microbial biomass samples, but widespread inclusion and analysis of controls still needs to be broadly implemented and reported 12, 57, 68. Below, we review the current information on sources of contamination and biases and provide recommendations for reducing these confounding factors when using microbiome assessments in diagnostic testing.

#### Sample collectors and technicians in the clinic and the laboratory

Professionals who collect and process samples may introduce their own microbial DNA into a sample (Figure 1), which is especially problematic when multiple individuals are collecting and processing samples for the same study or test. Most clinical protocols do not address microbial DNA contamination introduced from sample collectors and laboratory technicians, leading to the possibility that signals from individuals may override the signal from the biological sample if precautions and procedures are not put in place. Even the best trained sample collectors and laboratory technicians will introduce contamination into the samples; this is not an error on the part of the individual but now an appreciated signal within microbiome research. For example, sample collectors generally wear gloves and occasionally face masks to protect their health; however, DNA can be shed from unclean gloves, a mask, a lab coat, or the collector's unexposed skin 69 – all sources can now be detected using new HTS methods. To decrease the strength of these signals, all sample collectors and laboratory technicians should wear gloves, face masks, lab coats, and other appropriate clothing to prevent contamination from the technician 12. This problem is also not mitigated by the use of robotics, rather than human technicians. Automated robots produce more well‐to‐well contamination (cross‐contamination) across samples 70, which leads to specific batch and robot contamination. Additionally, bias can be introduced into samples of low microbial biomass through the technique applied by multiple sample collectors. Simpkins *et al* 71 showed that there was a significant difference in microbial species collected between technicians of varied experience (e.g. how hard a technician, doctor, or nurse presses down on a skin swab to collect a sample). Future research needs to fully characterise the contamination and bias introduced from sample collectors and laboratory technicians and understand how this can influence or alter microbial signatures in low microbial biomass samples.

#### The sampling and laboratory environments

Low microbial biomass samples are also influenced by the environment where they were collected (Figure 1) 60. Microbial profiles in different built and outdoor environments are unique, and even different laboratories with similar technicians and purposes have unique profiles 10, 13. Additionally, the same lab can have different contaminant profiles that change according to the season, year, or technician 13. For example, indoor environments, such as hospitals, resemble a microbial profile similar to that of the inhabiting individual(s) 72, 73. Despite this, the exact mechanisms that result in environmental contamination in low biomass samples are poorly understood. It is likely that the majority of microbes in a sampling environment exist in the air, in bioaerosols or on surfaces.

#### Reagents and equipment from sampling and laboratory processes

Low microbial biomass samples are highly susceptible to contaminating DNA present in reagents and equipment used for sample collection, DNA extractions, and library preparation (Figure 1). It is well recognised that reagents and equipment, including ‘sterile’ water, contain microbial DNA 13, 48, 74, 75. Salter *et al* 10 demonstrated that different extraction kits showed a different amount and composition of microbial DNA, and even the ‘cleanest’ kits contain reliable DNA signatures. This becomes problematic for low microbial biomass samples as the contaminating microbes can overwhelm the signal from endogenous DNA content 76, 77. During amplification, the exogenous DNA becomes preferentially amplified, lowering the chance of observing the true microbial signal. Varying concentrations of input DNA can also increase the number of artefacts during DNA amplification, which has a significant impact on low microbial biomass samples. Chafee *et al* 11 showed that input DNA concentration biases low microbial biomass samples by increasing duplication levels, favouring AT‐rich sequences, and overall biasing population levels. Generally, low microbial biomass samples have low DNA input levels for amplification reactions, which results in over‐amplification of the template DNA with high levels of duplication 11, 78. This can significantly bias results, suggesting that a single species or genus, perhaps even a contaminant species, is more dominant in the sample than it truly is. Consequently, these factors cause significant issues when reconstructing microbial communities for diagnostic purposes, especially when attempting to identify and quantify the microbes present.

### Recommendations to avoid potential contamination and biases

Below, we discuss the current recommendations to avoid potential contamination and biases when attempting to introduce microbiome analysis into diagnostic testing. For a quick reference guide, the RIDE checklist is also available as minimum standards for low microbial biomass samples 12.

1. *Include controls from the sampling and laboratory environments, equipment, and reagents*

To detect environmental microbes, air samples of the collection room should be gathered. Additional controls should include swabs of the hospital room (e.g. seats, walls, benches) before collection of biological samples. During laboratory processes, EBCs (controls that are run in parallel to the samples during the extraction, but do not include sample DNA) and no‐template amplification controls (NTCs; amplification reactions without any extracted DNA) should be included with every extraction or amplification batch, respectively, to monitor DNA incorporated into the sample via laboratory reagents. Additional controls to monitor any tool, substance, or individual that comes in contact with the samples may also be required for specific cases. It should be noted that additional amplification or strategies (e.g. the introduction of carrier DNA) 79 may be required to detect contaminating DNA and should be performed if necessary. Additionally, extraction methods that are known to contain fewer contaminants and are optimised for low microbial biomass samples (e.g. Mo Bio PowerMag with a ClearMag bead 54) should be used. Contaminating DNA simply cannot be avoided and needs to be monitored to ensure it is not driving the signals present in the collected samples.

2. *Minimise the amount of microbial and human contamination being introduced into samples*

Currently, there are no established protocols to minimise the introduction of microbial and human DNA into low microbial biomass samples in a clinical setting. However, other fields have methods that could be adopted or modified here, including ancient DNA protocols or those utilised in levels of high biosecurity 12, 13. Generally, introducing contaminating microbial DNA could be decreased by wearing clothing that covers exposed skin, such as wearing face masks and gloves. This is similar to the techniques used in ultra‐clean labs 12, where technicians are required to wear full disposable body suits, shoe covers, face masks, a plastic visor, and multiple pairs of gloves to minimise the introduction of human and bacterial DNA. Human DNA, which can overwhelm microbial DNA of many low microbial biomass samples, can also be depleted using methods such as Benzonase 33. Additionally, DNA in reagents and on equipment can be minimised by irradiating reagents with ultraviolet radiation 80. As microbial DNA is ubiquitous, these strategies can aid in reducing the contaminating DNA profile relative to the biological one, but it is critical to understand that current research supports the idea that it cannot be completely eliminated and should be monitored for the best results.

3. *Consistency and randomisation*

Inconsistency in sample collection (e.g. differences in pressure/duration when swabbing skin) can introduce biases. To minimise these biases, the best practice is to reduce the number of sample collectors. However, this is not possible at some diagnostic testing sites, so the collection and processing of samples should be randomised between sample collectors and laboratory technicians to minimise any possible biases. Alternatively, additional controls may be needed to account for this bias. Furthermore, standardised training and explicit sampling and processing protocols should be implemented to ensure that samples are collected and processed as similarly as possible. In the laboratory, positive controls with non‐biological DNA fragments (i.e. mock communities) can be utilised to ensure that technician bias during processing is limited and technician‐specific contamination can be more readily detected 12; however, the user must be careful that these positive controls are handled with consideration to their microbial biomass level. For example, it would be ill advised to add DNA from a positive, high biomass control first before processing low biomass samples in the same batch.

4. *Use quantitative laboratory methods*

In the past, the successful acquisition of DNA from a sample was determined by the presence or absence of bands on a gel. However, this technique is not sensitive to the level of DNA present in many control samples 54. Quantitative methods, such as fluorescent probes (e.g. PicoGreen) or quantitative PCR, should instead be utilised to determine the total DNA present in both samples and controls 54, 56. This process verifies that the biological sample has more DNA than the controls. It is also recommended that low input samples should be sequenced at a higher depth to capture a sufficient number of unique sequences 11. Further research is still needed to examine how low copy number biases can be best avoided in low biomass research.

5. *Incorporating bioinformatics approaches to assess or remove contamination*

Recently, several bioinformatic methods have been developed to track and remove contaminating DNA that influences low microbial biomass samples. First, the limit of detection can be applied using positive and negative controls 54, as described above. Additionally, individual contaminating species can be tracked from their source (i.e. environmental controls, reagents, equipment, EBCs, NTCs, *etc*.) using their exact sequence 81 and then can be subsequently removed or identified using publicly available programs, such as SourceTracker 82 and Decontam 64. While programs can be used to remove or track contaminants, the procedures do not mitigate the requirement to monitor and examine contaminants during sample collection and laboratory processes.

## Conclusion

In the near future, the microbiome will become an important asset for diagnostics and treating human diseases. However, most pathology samples contain low numbers of microbial cells, which makes them difficult to extract, amplify, and analyse in a microbiome context. These low microbial biomass samples are more prone to contamination and biases from the sample collectors and laboratory technicians, environment, reagents, and equipment. To avoid misdiagnoses and incorrect treatments, clear procedures and guidelines are needed for pathologists and clinicians to mitigate contamination and biases. Several groups, such as ancient DNA and forensic researchers, have developed similar guidelines and protocols for analyses done in their field 80, 83, 84. Proper controls, methodological precautions, and appropriate analytical strategies will allow the benefits of microbiome analysis to be appreciated for diagnostic testing in the future.

## Author contributions statement

CAS and LSW conceptualised the review. CAS wrote the original manuscript. LSW and RE critically reviewed and edited all versions of the manuscript.
